# Plasticity in Single Axon Glutamatergic Connection to GABAergic Interneurons Regulates Complex Events in the Human Neocortex

**DOI:** 10.1371/journal.pbio.2000237

**Published:** 2016-11-09

**Authors:** Viktor Szegedi, Melinda Paizs, Eszter Csakvari, Gabor Molnar, Pal Barzo, Gabor Tamas, Karri Lamsa

**Affiliations:** 1 MTA-NAP Research Group for Inhibitory Interneurons and Plasticity, Department of Physiology, Anatomy and Neuroscience, University of Szeged, Szeged, Hungary; 2 MTA-SZTE Research Group for Cortical Microcircuits, Department of Physiology, Anatomy and Neuroscience, University of Szeged, Szeged, Hungary; 3 Department of Neurosurgery, University of Szeged, Szeged, Hungary; 4 Department of Pharmacology, Oxford University, Oxford, United Kingdom; ICM - Institut du Cerveau et de la Moelle épinière, France

## Abstract

In the human neocortex, single excitatory pyramidal cells can elicit very large glutamatergic EPSPs (VLEs) in inhibitory GABAergic interneurons capable of triggering their firing with short (3–5 ms) delay. Similar strong excitatory connections between two individual neurons have not been found in nonhuman cortices, suggesting that these synapses are specific to human interneurons. The VLEs are crucial for generating neocortical complex events, observed as single pyramidal cell spike-evoked discharge of cell assemblies in the frontal and temporal cortices. However, long-term plasticity of the VLE connections and how the plasticity modulates neocortical complex events has not been studied. Using triple and dual whole-cell recordings from synaptically connected human neocortical layers 2–3 neurons, we show that VLEs in fast-spiking GABAergic interneurons exhibit robust activity-induced long-term depression (LTD). The LTD by single pyramidal cell 40 Hz spike bursts is specific to connections with VLEs, requires group I metabotropic glutamate receptors, and has a presynaptic mechanism. The LTD of VLE connections alters suprathreshold activation of interneurons in the complex events suppressing the discharge of fast-spiking GABAergic cells. The VLEs triggering the complex events may contribute to cognitive processes in the human neocortex, and their long-term plasticity can alter the discharging cortical cell assemblies by learning.

## Author Summary

Many microscale features in the human neocortex—a part of the brain involved in higher functions such as sensory perception, generation of motor commands, spatial reasoning, and language—are closely similar to those reported in experimental animals commonly used in neuroscience, like mice. However, the human neocortical neurons also exhibit specializations only reported in our species. One such feature is the capacity of excitatory principal cells to elicit firing in local inhibitory interneurons with a single action potential via very strong excitatory synapses. It has been suggested that this feature has specifically evolved to enhance coordinated firing of neuronal ensembles in higher brain functions. However, it is unknown how these circuits are modified by learning. Therefore, we investigated how these very strong excitatory synapses are changed, and if their impact on the firing of local inhibitory neurons is altered by repetitive action potentials mimicking learning-related activity. By recording in human neocortical slices, we report that the strong excitatory synapses on interneurons exhibit robust activity-dependent long-term plasticity. The plasticity also regulates the discharge of local interneurons driven by these synapses. Although these specialized synapses have only been reported in the human neocortex, their plasticity mechanism is evolutionarily conserved. We suggest that the strong synapses with robust plasticity have evolved to enhance complex brain functions and learning.

## Introduction

Evolution has shaped the human neocortex producing microcircuit features that are specific to our species [1]. Neuronal density, ultrastructural features, and functional properties of neurons [2–4] reflect specific adaptations in the human neocortex to perform complex and fast signal processing [5–13]. A remarkable feature in the human neocortex is that single pyramidal cell (PC) action potentials (APs) are able to generate di- and polysynaptic GABAergic interneuron discharge known as complex events [10,11]. The events emerge from the activity of a small subset of excitatory connections forming very large glutamatergic excitatory postsynaptic potentials (VLEs), specifically to GABAergic interneurons in supragranular layers of the frontal, the temporal, and the prefrontal cortices [10,11]. Similar strong excitatory connections between individual neocortical neurons have not been found in nonhuman brains. Therefore, it has been proposed that the VLEs and the complex events participate in cortical information encoding in high order cognitive processes [10]. However, this would predict that these events are dynamically modulated by learning [14,15]. Yet, it is unknown whether the VLEs show use-dependent long-term plasticity, and if their specific modulation indeed affects the complex events. We hypothesize that the immense strength of VLEs is generated and regulated by common activity-driven synaptic long-term plasticity processes, and that they may occur in various different inhibitory interneuron types [16,17]. Alternatively, these connections could be hard-wired selectively in a specific, yet unknown, subset of postsynaptic GABAergic interneurons without prominent lasting plasticity in the adult neocortex [18,19].

We asked whether VLEs show activity-induced long-term plasticity, and if their selective modulation had impact on the local complex events. By performing triple and dual whole-cell recordings of synaptically connected identified neurons, we found that VLEs exhibit metabotropic glutamate receptor (mGluR)-dependent long-term depression (LTD) that converts them to common weak excitatory postsynaptic potential (EPSP) connections. In addition, this alters the neocortical complex events suppressing the cell assemblies activated by the PC. Thus, the VLEs occur in various interneuron types, and their occurrence is regulated by the synapse’s activity history. To the best of our knowledge, this is the first study reporting synaptic plasticity in human neocortical interneurons.

## Results

### VLEs Occur in GABAergic Interneurons

**Fig 1:**
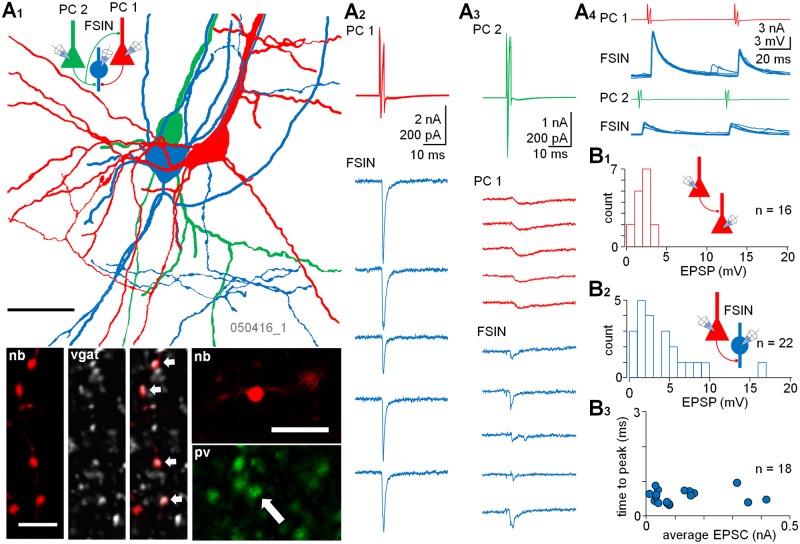
A subset of human neocortical PCs innervate FSINs with VLEs. (A) Triple whole-cell recording demonstrating the rich glutamatergic connectivity in the human neocortex layers 2–3 (L2–3). Two PCs (PC1 red and PC2 green) in L2–3 synaptically excite the same FSIN (blue). (A1) Partial reconstruction of the cells with color-coding presented in the schematic inset. Scale 25 μm. Confocal images illustrate positive immunoreactions of the neurobiotin (nb, Cy3)-filled interneuron axon boutons for vgat (Cy5, arrows in merged image) and pv (Alexa488, arrow). Scales 5 μm. (A2–3) Sample traces show presynaptic spikes (superimposed) and postsynaptic currents in the synaptic connections (cells voltage clamped at −60 mV). PC1 generates large monosynaptic EPSC in the interneuron (A2), whereas PC2 evokes small EPSC in the same cell (A3). In addition, PC2 is synaptically connected to PC1. The EPSCs from the PCs show fast kinetics in the interneuron, whereas EPSC in the PC–PC connection is slow. (A4) The two glutamatergic inputs to the FSIN show very different amplitude EPSPs and distinct paired-pulse ratios in current clamp (at Em –69 mV). (B) Histograms show the distribution of average EPSP amplitude (1 mV bin, failures excluded) in 16 identified L2–3 PC–PC pairs (B1) and in 22 PC–FSIN pairs (B2). (B3) The very large and the small amplitude EPSCs from PCs to FSINs show similarly fast time-to-peak time. Values are average EPSCs (of at least five) from individual pairs. The underlying data are shown in S1 Data.

By performing triple and dual whole-cell recordings from identified L2–3 human neocortical neurons, we found that some fast-spiking GABAergic interneurons (FSINs) receive glutamatergic input from individual afferent PCs showing VLEs (Fig 1A and 1B). Simultaneous recording from three neurons demonstrated that a fast-spiking GABAergic cell can receive VLEs (average amplitude 9.60 ± 0.20 mV, showing no failures) from one L2–3 PC and small amplitude glutamatergic EPSPs (average 3.29 ± 0.12 mV, showing no failures) similar to EPSPs between PCs from another PC [10]. Recordings from 21 synaptically connected PC–FSIN pairs revealed vast differences in the single AP-evoked EPSP amplitudes between the pairs (Fig 1B, S1 Table, S1 Data). In FSINs, the EPSP averages showed a range from 0.62 mV to 16.49 mV, with nonparametric distribution (failures excluded, evoked with 10 s interval at Em −68.5 ± 1.4 mV, *n* = 21, Shapiro-Wilk test). Despite the amplitude difference, the excitatory postsynaptic currents (EPSCs) in FSINs similarly exhibited fast time-to-peak kinetics (0.59 ± 0.04 ms, *n* = 18) (Fig 1B, S1 Data), indicating that the amplitude variability is unlikely to derive from different electrotonic filtering of the glutamatergic synaptic inputs. Likewise, distribution of the average EPSP amplitudes in PCs to non–fast-spiking interneuron (non-FSIN) pairs showed nonparametric distribution with a range from 0.7 mV to 6.9 mV (failures excluded, at Em −70.3 ± 1.5 mV, *n* = 9, Shapiro-Wilk test) (S1 Table). Thus, VLEs are not occurring solely in FSINs, but are exhibited in various types of GABAergic neurons including fast- and non–fast-spiking cells. On the contrary, PC–PC connections showed parametric distribution of average EPSPs with small amplitude (2.01 ± 0.02 mV at Em −69.4 ± 1.8 mV, failures excluded, Shapiro-Wilk test, *n* = 16) (Fig 1B, S1 Table, S1 Data). The interneuron EPSPs were defined as VLEs when their average (failures excluded) was larger than mean + 2 x standard deviation (SD) of the EPSPs in PC–PC connections (4.21 mV, failures excluded) in baseline conditions (mean ± SD = 2.01 ± 1.10 mV, *n* = 480 in 16 cells). The postsynaptic interneurons in the triple and paired recordings were immunohistochemically confirmed positive for vesicular GABA transporter (vgat+) (*n* = 31). Cells that in addition were immunopositive for parvalbumin (pv+) showed rapid axon currents (spike inward current width [SW] of 0.43 ± 0.02 ms, *n* = 11) characteristic of the FSINs [10,11]. The pv+ cells, together with vgat+ interneurons showing similar fast spike kinetics (SW 0.49 ± 0.02 ms, *n* = 11), but with nonconclusive or untested pv reaction, were considered FSINs (*n* = 22). Ten FSINs were further identified as putative basket cells by their axon morphology [10]. The non–fast-spiking vgat+ interneurons and the PCs had significantly longer spike kinetics with SW of 0.96 ± 0.04 ms (*n* = 9) and 1.18 ± 0.06 ms (*n* = 16), respectively (*p* < 0.01 between all groups, ANOVA with Tukey’s posthoc test) [13]. The non-FSINs with intact soma were immunohistochemically tested for somatostatin (sst) for further identification of the cells [20]. Detailed results on the EPSPs excluding and including failures, the EPSCs, and the immunohistochemical reaction analyses with cell type identification are shown in S1 Table. In all potential connections tested between neurons (*n* = 1,056), we found (including connections lost during baseline) a monosynaptic response in 11.0% of cases. Success rate for identified PC–FSIN pairs was 4.0%, PC–non-FSINs connections: 1.9%, PC–PC pairs: 1.1%, and FSIN–PC connections: 3.8%, showing similar or slightly lower connectivity rates than reported in the rodent neocortex L2–3 [21–23].

### VLEs in FSINs Are Subject to Group I mGluR-Dependent LTD

**Fig 2:**
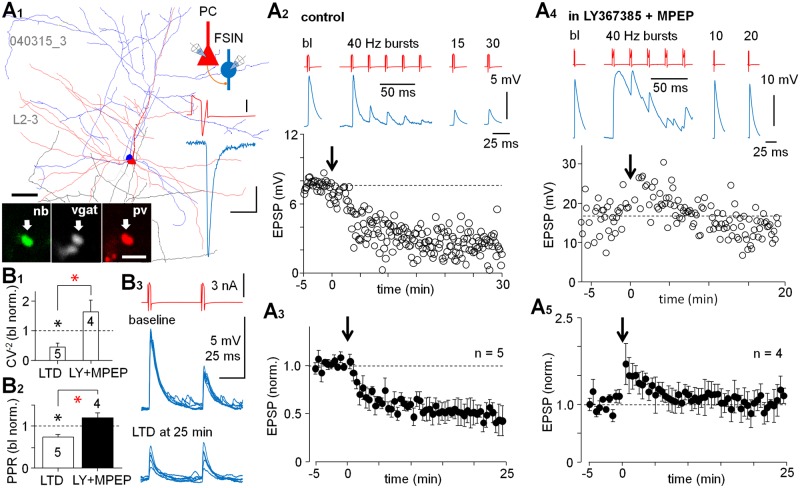
Single fiber connections to FSINs with large EPSP show group I mGluR-dependent LTD. (A) Paired recordings from synaptically connected layer 2–3 PCs and FSINs with large amplitude EPSCs/EPSPs show LTD. The LTD is generated by the presynaptic PC firing 40 Hz bursts (5 pulses, 40 times), while the postsynaptic cell is at Em. (A1) Partial reconstruction of one recorded PC (red, axon orange)–FSIN (blue, axon light blue) pair with large EPSCs/EPSPs. Scale 50 μm. L2–3 indicates layer 2–3. Schematic shows experimental design and color-coding for the cells and traces. Confocal micrographs illustrate vgat+ and pv+ axon bouton of the FSIN filled with neurobiotin (nb, scale 2 μm). A PC spike and averaged EPSC (5 at −60 mV) in the cell pair below. Scales 1 nA and 100 pA/5 ms. (A2) Single AP-evoked EPSP amplitude (interval 10 s) in the same experiment at baseline and following the PC 40 Hz burst firing (arrow at 0 time point). The afferent single fiber burst firing induced LTD (at 20−25 min *p* < 0.001, paired *t*-test). The EPSPs (blue, average of 5 at Em –62 mV) and presynaptic cell spikes (red) at different time points and one 40 Hz burst illustrated on top. The FSIN is at Em during the recording. (A3) Mean ± standard error of the mean (s.e.m.) in five PC–FSIN pairs with large EPSP (5.85 ± 0.59 mV at baseline, showing no failures) show prominent LTD (30 s bin, baseline-normalized, *n* = 5 pairs, *p* < 0.01, Wilcoxon test). (A4) The LTD requires group I mGluRs. A PC–FSIN pair with large EPSP in the presence of group I mGluR blockers LY367385 (100 μM) and 2-Methyl-6-(phenylethynyl)pyridine hydrochloride (MPEP, 25 μM) (applied 5 min before the bursts indicated by arrow). The EPSPs (blue, at Em −67 mV) and presynaptic cell spikes (red) shown on top. The FSIN is at Em during recording. (A5) Mean ± s.e.m. of similar PC–FSIN pairs with large EPSPs (8.42 ± 2.83 mV in baseline, showing no failures) in four experiments (30 s bin, baseline-normalized). The underlying data are shown in S2 Data. (B) EPSP analyses indicate presynaptic LTD. (B1) LTD (*n* = 5) is associated with an increased ratio of the EPSP amplitude SD/mean illustrated here as decreased baseline-normalized CV^−2^ (mean ± s.e.m. black asterisk, *p* < 0.05, Mann-Whitney test). Red asterisk compared with the non-LTD experiments (*n* = 4) (*p* < 0.05, Mann-Whitney test). (B2) Likewise, the EPSP amplitude PPR (1^st^ versus 2^nd^ EPSP) is reduced in the LTD experiments (black asterisk, *p* < 0.05, Mann-Whitney test), but not in the presence of group I mGluR blockers. Red asterisk indicates significance between the groups (*p* < 0.05, Mann-Whitney test). Baseline-normalized time window is 20–25 min after afferent bursts. The data are available in S3 Data. (B3) Sample traces from one experiment above showing the PC firing (paired-pulse 50 ms)-evoked EPSPs in the FSIN during baseline and in LTD.

We asked whether glutamatergic connections to interneurons showed long-term plasticity akin to that reported in the rodent cortex [24,25]. To test this, we performed experiments applying high frequency bursts of APs (5 APs at 40 Hz, x 40 with 0.5 s interval) in the presynaptic PC after a baseline of EPSPs (at least 5 min, but less than 10 min, analyzed including failures) [26,27]. Postsynaptic FSINs (SW 0.40 ± 0.02 ms) were held in resting membrane potential in current clamp (–67.6 ± 2.2 mV, *n* = 9) (Fig 2). First, we tested VLEs (average in baseline 5.85 ± 0.59 mV, did not show failures, *n* = 5) in control conditions and found that the afferent PC bursts firing generated an LTD of the EPSPs (amplitude to 0.52 ± 0.02 of baseline at 20–25 min after 40 Hz bursts, *n* = 5 cells, *p* < 0.01, Wilcoxon test) (Fig 2A, S1 Fig, S2 Data, S7 Data). The LTD was associated with a reduced paired-pulse EPSP ratio (1^st^/2^nd^ EPSP amplitude with 50 ms interval) to 0.74 ± 0.06 of baseline (*p* < 0.05, Mann-Whitney test, baseline mean 1.41 ± 0.22) and a decrease in the EPSP amplitude CV^−2^ (1/squared coefficient of variation) value (to 0.44 ± 0.12% from baseline, *p* < 0.05, Mann-Whitney test) (S3 Data), indicating presynaptic site of depression [28]. The results on the paired-pulse ratio (PPR) and altered EPSP amplitude variation by LTD are summarized in histograms in Fig 2B. Likewise, corresponding experiments in occasional PC–non-FSIN pairs with VLEs (*n* = 2, averages in the baseline including failures 5.81 mV and 6.89 mV, failure rates 0% and 13%, respectively) showed that single fiber burst firing can also generate LTD in some non-FSINs (S2 Fig).

We next tested whether LTD in VLEs requires group I mGluRs as various forms of long-term plasticity in glutamatergic synapses to FSINs depend on these receptors in the rodent cortex [24,29–32]. We studied four PC–FSIN pairs with VLEs (average in baseline 8.42 ± 2.83 mV at Em −69.5 ± 2.1 mV, did not show failures, *n* = 4) as above, but in the presence of LY367385 (100 μM) and 2-Methyl-6-(phenylethynyl)pyridine hydrochloride (MPEP, 25 μM). Thus, LTD was blocked in the VLE connections (Fig 2A) with no significant change in the amplitude (1.03 ± 0.04 of baseline at 20–25 min, *n* = 4 cells, Wilcoxon test), PPR (1.21 ± 0.11 of baseline, baseline mean 1.07 ± 0.17) or 1/CV^2^ (1.63 ± 0.39 of baseline) (Mann-Whitney test) (Fig 2B, S2 and S3 Data). To conclude, PC–FSIN connections with large EPSPs show activity-driven LTD, which requires group I mGluRs.

### LTD by Single PC Bursting Is Specific to VLEs

**Fig 3:**
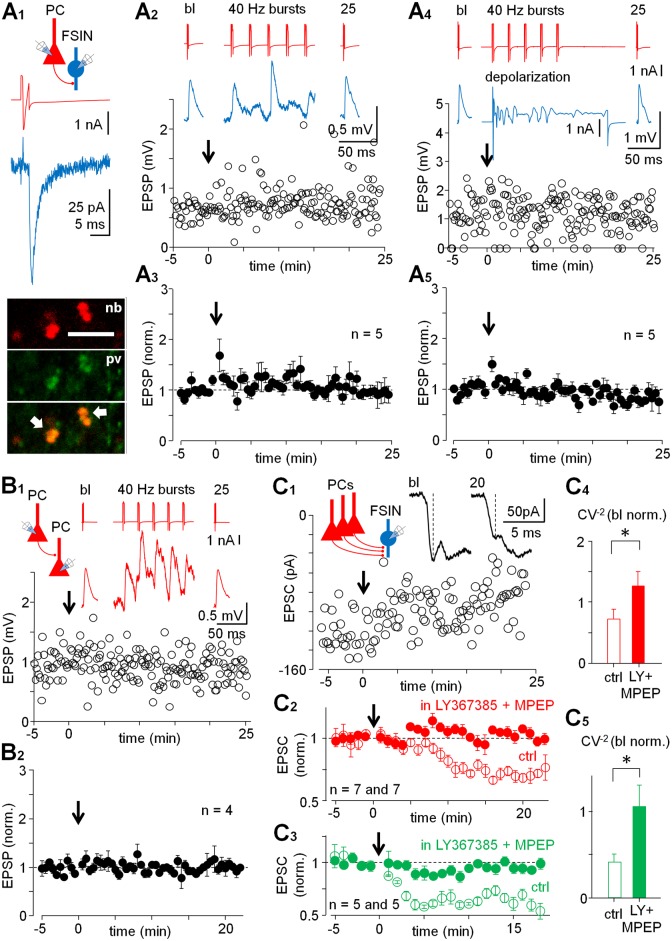
LTD fails in weak single-fiber PC-FSIN connections, but is generated by coactivity of multiple glutamatergic fibers. (A) LTD fails in PC–FSIN pairs with small EPSP. (A1) Schematic shows experimental design. A presynaptic PC spike (red) with postsynaptic EPSCs (blue, average of 5 at −60 mV) in one recording and confocal micrographs of the FSIN axon (nb, neurobiotin) with pv+ boutons (scale 5 μm, arrows point colabeling in merged image). (A2) One PC–FSIN pair with the EPSPs in baseline and after the afferent cell 40 Hz bursts (arrow). Averaged EPSPs (5) at Em −72 mV on top at different time points and a 40 Hz burst. Postsynaptic cell is at Em (current clamp) during the recording and the bursts. (A3) Mean ± s.e.m. (30 s bin, baseline-normalized) of similar experiment in five PC–FSIN pairs with small amplitude EPSPs (1.89 ± 0.43 mV in baseline with failures). (A4) Failure of the LTD in weak PC–FSIN connections is not due to insufficient postsynaptic depolarization. Plot shows EPSP in one PC–FSIN pair before and following the presynaptic bursts, now paired with FSIN depolarization beyond the firing threshold (see Methods). Averages of EPSPs (5 at Em −66 mV) and a 40 Hz burst with simultaneous depolarization (30 mV, 250 ms) in voltage clamp shown on top. (A5) Mean ± s.e.m. of five similar experiments with small EPSP (1.44 ± 0.22 mV in baseline with failures) PC–FSIN pairs (baseline-normalized, 30 s bin). The underlying data are shown in S4 Data. (B) Connections between PCs exhibit small amplitude EPSPs with no long-term plasticity when PC1 bursts, while PC2 is at resting membrane potential. (B1) EPSP amplitude in one experiment before and after the 40 Hz presynaptic cell bursts (arrow, postsynaptic cell at Em −78 mV). Averaged EPSPs (five at Em) shown on top with a 40 Hz burst, and a schematic showing the experimental design. (B2) Mean ± s.e.m. of baseline-normalized EPSPs (1.40 ± 0.30 mV in baseline with failures) in four PC–PC pairs as in *B1* (30 s bin) (S4 Data). (C) Activation of multiple afferent pathways to FSINs using extracellular stimulation reveals group I mGluR-dependent LTD in weak PC–FSIN synapses (S5 Data). (C1) One experiment with monosynaptic EPSC in FSIN (voltage clamped at −60 mV) at baseline and following the 40 Hz bursts applied to the stimulation pathway (arrow at 0 time point). Inset traces (averages of 5) show evoked EPSCs in baseline and in LTD. The monosynaptic component is indicated by dotted vertical line. Schematic shows experimental design. (C2) Mean ± s.e.m. of seven baseline-normalized experiments as in *C1* showing the LTD in control conditions (open symbols, *p* < 0.001, paired *t*-test) and blockade of the LTD in experiments with LY367385 (100 μM) and MPEP (25 μM) (solid symbols, *n* = 7, paired *t*-test). (C3) Generation of group I mGluR-dependent LTD by 40 Hz stimulation is conserved in mammalian neocortex occurring also in rat FSINs. Multiple fiber extracellular stimulation with LTD in rat L2–3 somatosensory cortex FSINs. Open symbols show experiments in control conditions (*n* = 5, *p* < 0.01) and solid symbols in the presence of LY367385 (100 μM) and MPEP (25 μM) (*n* = 5) (Wilcoxon test). Blockers for glutamate *N*-methyl-D-aspartatereceptors (NMDARs) (DL-2-Amino-5-phosphonopentanoic acid; DL-APV, 100 μM) and GABA_A_Rs (PiTX, 100 μM) were present in *C1–C3*. (C4-C5) Likewise, LTD of the EPSCs in both species is associated with an increased amplitude SD versus the mean. Data shows decreased CV^−2^ (baseline-normalized at 20 min after 40 Hz) in LTD in control conditions, but not when LTD is blocked in the presence of group I mGluR blockers (*p* < 0.05 between groups, Mann-Whitney test).

In contrast to VLEs, the PC–FSIN pairs with small EPSPs (average in baseline with failures 1.89 ± 0.43 mV at Em −69.2 ± 3.5 mV, failure rate 11.2 ± 9.5%, SW 0.48 ± 0.04 ms, *n* = 5) failed to show lasting plasticity following the 40 Hz bursts (amplitude 1.07 ± 0.06 of baseline at 20–25 min) (Fig 3A, S1 Fig, S4 Data). Likewise, no lasting plasticity was seen in any (paired *t*-test) of the three vgat+ non-FSINs (SW 0.91 ± 0.07, *n* = 3) with small amplitude EPSP (2.08 ± 0.58 mV, *n* = 3) in similar experiments. Given that LTD in the VLEs was accompanied by stronger postsynaptic depolarization during the presynaptic spike bursts, we studied whether small amplitude EPSP connections would show plasticity if the postsynaptic FSIN was depolarized during the PC bursts. We reproduced experiments above with a separate set of PC–FSIN (SW 0.47 ± 0.03 ms, *n* = 5) pairs with small amplitude EPSP (average with failures 1.44 ± 0.22 mV, failure rate 11.0 ± 3.3%, *n* = 5), and paired presynaptic PC spike bursts with postsynaptic cell depolarization (20–30 mV, 250 ms steps from Em, see Methods for details) beyond the firing threshold (Fig 3A). This protocol also failed to generate long-lasting change in EPSPs in the PC–FSIN pairs (0.94 ± 0.04 of baseline at 20–25 min, *n* = 5, Wilcoxon test) (Fig 3A, S4 Data). Interestingly, a small but significant LTD was observed with this configuration in two (0.71 ± 0.10 and 0.83 ± 0.05 at 20–25 min compared to baseline, *p* < 0.05 for both cells, paired *t*-test) of three individual PC–non-FSIN (vgat+) pairs tested. Finally, we studied the synaptic connections between L2–3 PCs applying presynaptic 40 Hz bursts while the postsynaptic cell was at resting membrane potential. The PC–PC pairs were connected with small amplitude EPSPs (average with failures 1.40 ± 0.30 mV at Em −65.9 ± 5.4 mV, failure rate 4.2 ± 3.2%, *n* = 4), and the 40 Hz bursts failed to generate lasting plasticity in the EPSP (1.01 ± 0.05 of baseline at 20–25 min, *n* = 4 cells, Wilcoxon test) (Fig 3B, S4 Data), possibly because LTP and LTD in human PCs require strong postsynaptic depolarization for either activation of glutamate NMDA receptors or L-type voltage-gated calcium channels [7]. Input resistance in the plasticity recordings showed small increase to 1.09 ± 0.01 (baseline-normalized) at 20–25 min from baseline (*n* = 26, *p* < 0.01, *t*-test) [33]. The results show that following just a single PC burst firing, LTD specifically occurs in large EPSPs between PCs and interneurons, and not in other investigated synaptic connections.

### Co-activation of Many Weak Glutamatergic Synapses Evokes mGluR-Dependent LTD in Fast-Spiking Interneurons

Because studies in rodents have reported LTD in glutamatergic synapses to cortical interneurons either by chemical or strong synaptic activation of group I mGluRs, we studied whether FSINs with weak excitatory inputs showed the LTD when multiple glutamatergic fibers were simultaneously activated (Fig 3C) [27,29,34]. Evoking compound EPSCs from many small glutamatergic inputs with extracellular electrical stimulation (see Methods), we applied 40 Hz bursts to the glutamatergic pathway as above after baseline (at least 5 min, but less than 10 min). Focusing the EPSC analysis in the FSINs on the monosynaptic component of the current (see Fig 3C) [35,36] we found that the 40 Hz burst stimulation resulted in LTD (EPSC 0.72 ± 0.02 from baseline at 20 min, *n* = 7 cells, *p* < 0.001, *t-*test). Blockers for glutamate *N*-methyl-D-aspartatereceptor (NMDARs) (DL-2-Amino-5-phosphonopentanoic acid [DL-APV], 100 μM) and GABA_A_Rs (PiTX, 100 μM) were present in the experiments. The EPSC amplitude LTD was accompanied by decreased CV^−2^ (baseline-normalized to 0.72 ± 0.16%, *n* = 7, *t*-test). This LTD was blocked in experiments with group I mGluR antagonists LY367385 (100 μM) and MPEP (25 μM) (*n* = 7, *p* < 0.05, *t-*test) (Fig 3C, S5 Data). The FSINs in these extracellular stimulation experiments showed narrow SW 0.62 ± 0.04 ms, *n* = 14. Accordingly, we reproduced these experiments with rat glutamatergic fibers to FSINs in L2–3 (SW 0.64 ± 0.06 ms, *n* = 10) and confirmed LTD (EPSC 0.64 ± 0.04 from baseline at >15 min, *n* = 5, *p* < 0.01, Wilcoxon test) and its blockade with the group I mGluR antagonists (EPSC from baseline 0.95 ± 0.5, *n* = 5, Wilcoxon test) (Fig 3C, S5 Data). The EPSC amplitude in LTD showed reduced CV^−2^ (baseline-normalized to 0.41 ± 0.09%, *n* = 5, Mann-Whitney test), but not when LTD was blocked with the mGluR antagonists (1.04 ± 0.24% of baseline at >15 min) (*p* < 0.05 between the groups at >15 min, Mann-Whitney test) (Fig 3C, S5 Data). We tested three of the recorded rat FSINs for pv immunoreaction and found them all positive. Thus, in both human and rat cortex, weak glutamatergic connections to L2–3 FSINs exhibit group I mGluR-dependent LTD if multiple glutamatergic inputs are activated simultaneously.

### LTD of VLEs Is Sufficient to Modify Network Activity

**Fig 4:**
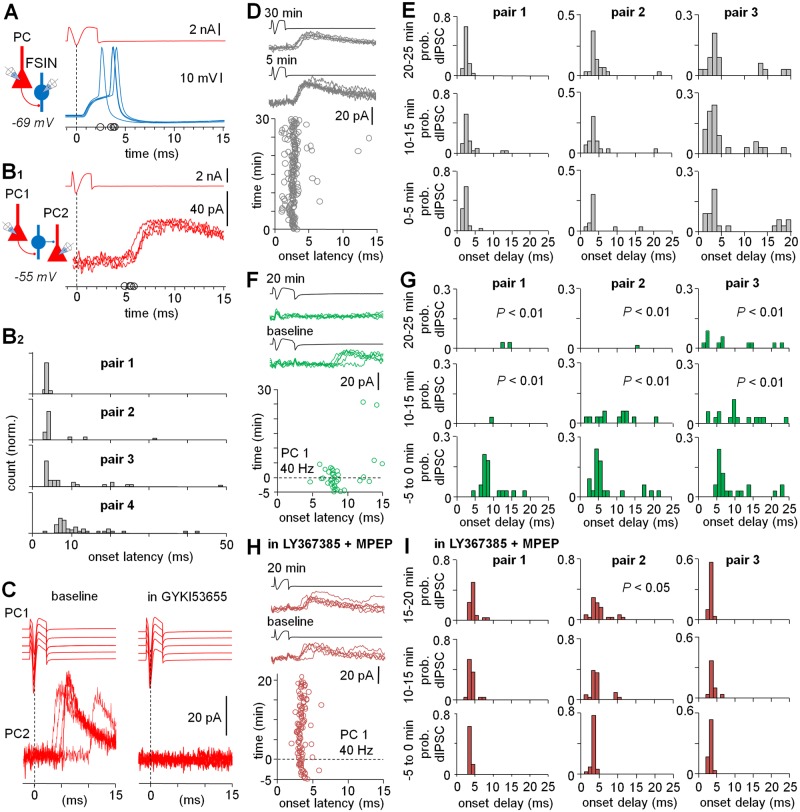
The LTD depresses discharge of FSINs in complex events. (A) Large EPSPs and APs in FSIN (blue) with short 3 to 5 ms delay elicited by single PC spike (red, peak indicated by vertical line). The figure shows four consecutive cycles with 10 s interval in one vgat+ and pv+ FSIN at Em (−69 mV) (note short AP duration, the positive peaks are indicated in the abscissa). Schematic shows experimental setting. (B) Single PC spike triggers disynaptic GABAergic currents in the layers 2–3. Dual whole-cell PC recordings (voltage-clamp) show that single PC1 APs trigger dIPSCs in PC2 with high probability and short delay. Schematic shows experimental design. (B1) Traces show consecutive events (4) in one experiment. The dIPSC onsets are marked in abscissa. (B2) Histograms (ordinates normalized and show from 0 to 1) illustrate delay distribution of the first dIPSC onset in four experiments (each 6–9 min, indicated as pairs 1–4) in control conditions. Most evoked dIPSCs are phase-locked to the presynaptic PC spike with <10 ms delay with obvious moderate variability of the mode of delay between experiments (S6 Data). (C) The dIPSCs are generated by glutamatergic excitation. Sample traces show five consecutive dIPSCs between PC1 and PC2 in baseline conditions and the blockade with AMPAR blocker GYKI53655 (25 μM) (see S4 Fig). (D–E) The single AP-evoked dIPSCs between PCs are stable over a long period (S6 Data). (D) Raster plot illustrates timing of dIPSC onset (in PC2) evoked by an AP in the presynaptic PC (PC1) in one 30 min experiment. Consecutive (6) presynaptic spikes and dIPSCs at different time points shown on top. (E) Three experiments (pairs 1, 2, and 3) as in *D* (pair 1), illustrated with histograms showing the dIPSC onset delay in different time windows (0–5 min, 10–15 min, and 20–25 min). The dIPSC probability and delay are stable for at least 30 min (Chi-square test). (F–G) dIPSCs show LTD after presynaptic PC 40 Hz burst firing (S6 Data). (F) 40 Hz spike bursts in the presynaptic PC (similar to the LTD experiments in Fig 2) induce LTD of the dIPSCs. Raster plot shows dIPSCs in one paired PC recording. After baseline, the 40 Hz spike bursts in PC1 (at 0 time point, dotted horizontal line) induce permanent depression of the dIPSC occurrence. Traces illustrate the presynaptic cell spike and the dIPSCs (6) at baseline and the absence of dIPSCs after 20 min. (G) Three similar experiments (pairs 1, 2, and 3) as in *F* (pair 1), showing the LTD of dIPSCs after the 40 Hz presynaptic bursts (BL −5 to 0 min) (*p* < 0.01, Chi-square test). (H–I) The LTD of dIPSCs is blocked with group I mGluR antagonists (S6 Data). (H) Similar experiment as in *F*, but in the presence of LY367385 (100 μM) and MPEP (25 μM). Traces on top show the pre- and disynaptic currents at baseline and 20 min after the 40 Hz bursts. (I) Three experiments as in *H* (pair 1) illustrated with dIPSC onset delay histograms at the baseline (−5 to 0 min) and at two time windows after the 40 Hz presynaptic bursts.

Given that interneuron–PC connections with VLEs have been proposed to be essential in generation of the neocortical complex events, we studied whether the LTD in these connections would selectively modify network activity. First, we confirmed that single PC AP-evoked VLEs in the FSIN as well as in the non-FSIN elicited firing of these interneurons from the resting membrane potential [11]. We found that VLE-evoked postsynaptic spikes in a FSIN (Em −69 mV) typically followed with a short 3–5 ms delay (Fig 4A) [10], whereas in a non-FSIN, (Em −69 mV) the spikes showed long delay with large jitter (S2 Fig). Similarly, whole-cell recordings between identified PCs revealed disynaptic GABA_A_R-mediated inhibitory currents (dIPSCs) in complex events elicited by a single AP (interval 10 s) (Fig 4B) [10,11]. The dIPSCs occurred with short delay (6.23 ± 0.72 ms, *n* = 16 pairs) and high probability (0.70 ± 0.05, *n* = 16 pairs in baseline conditions) (S6 Data). The dIPSCs showed longer and more variable delay to the presynaptic spike than monosynaptic GABA_A_R-mediated inhibitory currents (monIPSCs) from FSINs (0.96 ± 0.10 ms, *n* = 9, *p* < 0.001, *t-*test) (SW 0.48 ± 0.03 ms, *n* = 9) (S3 Fig, S9 Data). In addition, dIPSCs were blocked by the glutamate AMPAR blocker GYKI53655 (25 μM) (*n* = 3, *p* < 0.001, Chi-square test) (Fig 4C, S4 Fig, S6 Data). The evoked dIPSC amplitudes (averages excluding failures in all plasticity recordings 32.1 ± 3.7 pA, *n* = 12) were similar to monIPSCs (35.2 ± 5.6 pA, *n* = 9, *t*-test) (S5 Fig, S10 Data), indicating that these early complex event inhibitory currents (IPSCs) were generated by a single FSIN. The dIPSCs and the monIPSCs were recorded at −55 mV. The dIPSCs were detected in 3.0% of all potential connections tested (*n* = 1,056).

To determine whether plasticity modified this network, we performed long recordings from PC pairs showing that the probability and delay of the dIPSCs were stable for at least 30 min (*n* = 3) in normal conditions (Fig 4D, 4E and S6 Data). However, if the 40 Hz burst firing was delivered in the presynaptic PC (similar to the VLE LTD experiments in Fig 2), the dIPSC occurrence (total in 25 ms from PC spike) rapidly and permanently attenuated after a baseline showing strong LTD (from 0.71 ± 0.04 in baseline to 0.14 ± 0.09 at 15–20 min, *n* = 3, *p* < 0.05, Chi-square test) (Fig 4F, 4G and S6 Data). Interleaved experiments in the presence of group I mGluR antagonists LY367385 (100 μM) and MPEP (25 μM) showed that LTD of dIPSCs was blocked in the presence of the group I mGluR antagonists (0.81 ± 0.10 in baseline and 0.88 ± 0.12 at 15–20 min, *n* = 3, Chi-square test) (Fig 4H, 4I and S6 Data). In conclusion, the results demonstrate that a single PC burst firing at 40 Hz elicits robust LTD in VLE connections to FSINs and causes suppression of phase-locked early dIPSCs between PCs. These two LTDs both require group I mGluRs. Thus, the results show that plasticity of VLEs in FSINs changes the activation pattern of the neurons discharging in supragranular layers during complex events.

## Discussion

Almost a decade ago, Molnar et al. [10] first reported that a subset of excitatory PC synapses in the human neocortex form VLEs in local GABAergic interneurons in supragranular layers. These strong connections specifically from PCs to inhibitory interneurons have since been reported in the frontal, prefrontal, parietal, and temporal cortices, where the VLEs often are suprathreshold, driving assemblies of inhibitory interneurons to fire after a single PC spike [11,37]. Similar strong connections between two individual neocortical neurons have not been found in nonhuman species [37–39]. Analyses of large datasets from rodent visual and somatosensory cortices have revealed that neurons in local networks are not randomly connected, but specific local connectivity patterns exist between neuron types, and the strongest excitatory synapses control local network activity [40–42]. This suggests that there is a skeleton of strong connections in the network that dominates the activity [40]. Therefore, it has been proposed that the VLE connections in the human might be important in generating neocortical cell assemblies and be involved in higher cognitive functions [11]. However, until now it had remained unknown how neuronal plasticity regulates these connections and whether their selective modulation indeed alters discharge of neuronal assemblies in the human neocortical network activity.

Our result that the VLEs occur specifically in human interneurons, and not in between PCs, is consistent with previous studies [10,11]. In addition, we demonstrate that VLEs are generated in different interneuron subpopulations, including fast-spiking (such as basket cells) and non–fast-spiking supragranular vgat+ neurons. Furthermore, single GABAergic interneurons receive both VLEs and more common small EPSP connections from layer 2–3 PCs. Indeed, anatomically identified L2–3 basket cells show huge variability in strength of PC inputs. It is likely, although we do not directly demonstrate it here, that a single presynaptic L2–3 PC cell evokes VLEs and small EPSPs in different L2–3 postsynaptic interneurons showing synapse specificity. A comprehensive recent study in rodent demonstrated that certain connectivity patterns between neurons are repeated in the neocortex across different regions [39]. The strong VLE connection, occurring between PCs and L2–3 inhibitory interneurons in various neocortical regions is one such feature, and probably specific to the human neocortical microcircuits.

We show that strength of VLE connections is controlled by their activity history. A common form of mGluR-dependent LTD characterized in the extracellular stimulation experiments of glutamatergic synapses in the rodent cortex [24] converts the VLEs to small amplitude EPSPs. This LTD suppresses VLE synapses through a presynaptic mechanism most likely controlling the vesicular transmitter release as indicated by the changes in the EPSP amplitude PPR and the coefficient of variation in the depressed synapses [37]. The LTD of VLEs in FSINs, many of which are also pv+, involves the group I mGluRs. In the rodent cortex, group I mGluRs have been shown to play a central role in long-term plasticity processes including presynaptic LTD and LTP [24,29–31,34,43–46]. Our results from the group I mGluR-dependent LTD in FSINs in the human and the rodent neocortex indicate that this is an evolutionarily conserved plasticity mechanism for controlling the fast-spiking interneuron activity in the mammalian brain.

Interestingly, we report that in a non–fast-spiking cell, LTD was not blocked by the group I mGluR antagonists (see S2 Fig). In addition, we found significant LTD in two of three PC–non-FSIN connections with small EPSP when the postsynaptic cell was depolarized during PC bursts. These suggest that there may be various LTD forms in human cortical interneuron synapses [24,25], and that non-FSINs may exhibit different LTD mechanism than the FSINs, possibly depending on postsynaptic depolarization. Indeed, many synapse-specific properties including long-term plasticity have been reported in glutamatergic fibers in the rodent cortex [21,32,47–50]. Correspondingly, synapses originating from the same PC in the human neocortex may exhibit distinct long-term plasticity, depending upon the postsynaptic target cell; and different activity patterns may be required for plasticity in the synapses [24,50]. In this study, we have used elevated extracellular calcium (3 mM) to increase the stability of disynaptic IPSCs in baseline conditions. However, compared to recordings with 2 mM extracellular calcium, this modification is unlikely to strongly affect the high-frequency firing-evoked synaptic release and the long-term plasticity in FSIN synapses: the probability of synaptic release in VLEs in FSINs is already very high at 2 mM Ca^2+^, and only slightly modulated by further increase of calcium [37]. Yet, as demonstrated in the recent study by Molnar et al. [37], the VLE release probability can markedly decrease when extracellular calcium is reduced from 3 mM to 1.5 mM, which is considered lower range of the cerebrospinal fluid total calcium level in physiological conditions [51]. Therefore, it is also possible that in calcium concentrations close to 1.5 mM, the PC firing pattern used in this study may not be sufficient for such a robust LTD as reported here in the FSINs. Importantly, some non-FSINs show VLEs with low presynaptic release probability even in 3 mM calcium, as indicated by the large EPSP amplitude coefficient of variation. In these synapses, extracellular calcium modulations may have even stronger effects on the short- and long-term plasticities than in the FSINs.

Strikingly, in the human neocortex, the activity of a single PC is sufficient to trigger mGluR-dependent LTD in the VLE connections, but not in weak glutamatergic pathways. The level of the postsynaptic depolarization does not explain the failure of LTD in small EPSP connections to fast-spiking cells, and a potential explanation is that there is insufficient activation of the postsynaptic group I mGluRs [34,43]. The connections with VLEs are likely to release more glutamate and activate the critical mGluRs [52–54]. The hypothesis on strong glutamate release is supported by our finding that the small EPSP connections were unable to generate LTD by single fiber activity, but they showed the mGluR-dependent plasticity when multiple fibers were activated simultaneously with local extracellular stimulation [55]. Indeed, a recent study revealed that human neocortical PC–FSIN synapses with VLEs have more transmitter vesicle release sites, although the glutamate release quantal size is similar compared to synapses in rat neocortex [37]. This indicates multivesicular release in synapses with VLEs, and it is interesting to speculate that the conversion to common small EPSPs via the presynaptic LTD might reflect their transformation from a multivesicular release site to a single vesicle-releasing synapse.

Although a link between very large excitatory synapses and human cortical complex events has been suggested earlier [10,11], a relation between their selective modulation and complex events had not been directly demonstrated until this study. The LTD triggered by a single PC firing in the current conditions is specific to connections with VLEs and therefore provides a useful tool to test the relation between VLEs and neocortical network activity. Results here show that the VLE connections indeed trigger the complex events, and the LTD changes their temporal structure. The activation of individual fast-spiking interneurons was commonly observed in dual PC recording as disynaptic GABAergic currents with timing corresponding to the APs in fast spiking interneurons [11]. LTD of the VLE in FSINs and the suppression of dIPSCs in complex events were both induced by single PC burst firing, and they both required group I mGluRs. Some non-FSINs also exhibit VLEs and can show LTD, although these cells were unlikely to contribute to the GABAergic disynaptic currents investigated here: the dIPSCs occurred with short delay and high precision, whereas the non-FSINs show long and variable delay in their response to fire APs (see S2 Fig). However, the results of non-FSINs are based on small sample sizes, and should therefore be interpreted with caution.

In conclusion, the human neocortex is unique in many aspects, since its microcircuits show differences at the molecular, ultrastructural, and physiological levels compared to other mammalian species [1,3,56]. The capacity of the human neocortex to perform extraordinary and highly complex tasks may at least partly result from these microcircuit level specializations. We propose that VLEs with robust activity-induced plasticity and their contribution to neocortical cell assemblies may be crucial for higher cognitive functions and abstract mental abilities of the human brain. In addition, evidence in animal models suggests the involvement of group I mGluR-mediated plasticity in neocortical learning processes, and perturbation of the mGluR-dependent plasticity has been reported with mental decline [43]. Therefore, the human-specific microcircuit features may also be substrates for pathological processes resulting in cognitive decline and other neurological and neuropsychiatric dysfunctions that we as a species are vulnerable to [57–61].

## Materials and Methods

### Ethics Statement

All procedures were performed according to the Declaration of Helsinki with the approval of the University of Szeged Ethical Committee and Regional Human Investigation Review Board (ref. 75/2014).

### Electrophysiology and Analysis

Human neocortical slices were derived from material that had to be removed to gain access to the surgical treatment of deep-brain tumors from the left and right frontal, temporal, and parietal regions with written informed consent of the patients prior to surgery. The patients were 10–85 y of age (mean ± SD = 50 ± 4 y), including 17 males and 14 females. The tissue obtained from underage patients was provided with agreement from a parent or guardian. The resected samples were cut from the frontal and temporal lobes of left or right hemisphere. Anesthesia was induced with intravenous midazolam and fentanyl (0.03 mg/kg, 1–2 lg/kg, respectively). A bolus dose of propofol (1–2 mg/kg) was administered intravenously. The patients received 0.5 mg/kg rocuronium to facilitate endotracheal intubation. After 2 min, the trachea was intubated and the patient was ventilated with O_2_/N_2_O mixture (a ratio of 1:2). Anesthesia was maintained with sevoflurane at monitored anesthesia care volume of 1.2–1.5. After surgical removal, the resected tissue blocks were immediately immersed in ice-cold standard solution containing (in mM): 130 NaCl, 3.5 KCl, 1 NaH_2_PO_4_, 24 NaHCO_3_, 1 CaCl_2_, 3 MgSO_4_, 10 D(+)-glucose, and saturated with 95% O_2_ and 5% CO_2_. Slices were cut perpendicular to cortical layers at a thickness of 350 μm with a microtome (Microm HM 650 V) and were incubated at room temperature (20–24°C) for 1 h in the same solution. Rat neocortical slices were prepared as described before [62]. Male Wistar rats were anaesthetized using halothane, and following decapitation (320 μm thick), coronal slices were prepared from the somatosensory cortex. The solution used during electrophysiology experiments was identical to the slicing solution, except it contained 3 mM CaCl_2_ and 1.5 mM MgSO_4_. Recordings were performed in a submerged chamber (perfused 8 ml/min) at approximately 36–37°C. Cells were patched using water-immersion 20× objective with additional zoom (up to 4x) and infrared differential interference contrast video microscopy. Micropipettes (5–8 MOhm) were filled with intracellular solution for whole-cell patch-clamp recording (in mM): 126 K-gluconate, 8 KCl, 4 ATP-Mg, 0.3 Na_2_–GTP, 10 HEPES, 10 phosphocreatine (pH 7.20; 300 mOsm) with 0.3% (w/v) biocytin. Current and voltage clamp recordings were performed with Mutliclamp 2B amplifier (Axon Instruments), low-pass filtered at 6 kHz (Bessel filter). Series resistance (Rs) and pipette capacitance were compensated in current clamp mode and pipette capacitance in voltage clamp mode. Cell capacitance compensation was not applied. Rs was monitored and recorded continuously during the experiments. The recording in voltage clamp mode was discarded if the Rs was higher than 25 ΩM or changed more than 20%.

In paired cell recordings, APs were generated in the presynaptic cell with brief (2–3 ms) suprathreshold depolarizing (60–70 mV) paired pulses (50 ms interval) in voltage clamp delivered every 10 s from −60 mV. Postsynaptic cells were at resting membrane potential in current clamp mode. In some cells with VLEs, the postsynaptic cell was hyperpolarized (up to −10 mV) with constant current to prevent the VLE from triggering an AP. The 40 Hz firing protocol was similarly applied in voltage clamp mode with series of 2–3 ms depolarizing pulses (5 pulses at 40 Hz, delivered every 0.5 sec 40 times), while the postsynaptic cell was held in current clamp resting membrane potential. In some experiments (Fig 3A, 4–5), the postsynaptic cell was depolarized in voltage clamp during presynaptic 40 Hz firing with a continuous step (20–30 mV, 250 ms). This elicited on average 2.2 postsynaptic spikes for 1^st^ presynaptic spike (in following 25 ms, *n* = 200 in 5 cells) of the 40 Hz train, and on average 0.80 postsynaptic spike probability for the 2^nd^–5^th^ PC AP.

Extracellular stimulation was applied with a concentric bipolar electrode (125 μm tip diameter, FHC Inc., US) positioned on L2–3. Paired pulse stimuli (50 μs, with 50 ms interval, intensity range from 20 to 300 μA) were delivered every 15 s with current isolator stimulator (Model DS3, Digitimer, UK). Compound EPSCs in Fig 3 were confirmed by observing less than 100 pA increases in the evoked EPSC amplitude when gradually increasing stimulation intensity.

### Data Analysis and Statistics

Data were acquired with Clampex software (Axon Instruments, US) at 20 kHz. EPSC/P, IPSC, action current duration, and the cell input resistance were analyzed off-line with p-Clamp software (Axon Instruments, US) and Spike2 (version 7.0, Cambridge Electronic Design, UK). Liquid junction potential was not corrected. EPSC amplitude and kinetics analysis in voltage clamp mode (time-to-peak from onset) was omitted when access resistance was higher than 25 MΩ. SW was calculated from the onset of inward action current till recovery to baseline holding level. Data for SWs were collected in the beginning of experiments when synaptic connections from all cells were briefly tested in voltage clamp mode. All data are presented as mean ± s.e.m. and when showing baseline-normalized EPSPs of many cells, the values were calculated from binned (30 s bin) data. In rare cases when cell-spiked and accurate EPSP amplitude data was not available, bin includes two instead of three data points. For statistical analysis, ANOVA with posthoc Tukey’s test and *t*-test were used for data with normal distribution (Shapiro-Wilk test) and sample sizes larger than *n* = 6. Chi-square test was used for categorical variables (occurrence of dIPSC in 25 ms time window from PC spike). Otherwise, Mann-Whitney U-test (unpaired) and Wilcoxon Signed Rank Test (paired) were used. EPSP amplitude in individual plasticity experiments was tested with paired *t*-test comparing data points in 5 min baseline and an equal time window at 20–25 min following the presynaptic bursts, unless stated otherwise (in some shorter experiments in the last 5 min of the recording). Correlation was determined with Pearson’s r-test. Differences were accepted as significant if *p* < 0.05. Failures were included in the EPSP mean values (in binned data) in plasticity analysis.

### Drugs

DL-APV, GYKI53655, LY367385, MPEP, and picrotoxin (PiTX) were applied via bath and purchased from Sigma Aldrich (Hungary).

### Cell Visualization and Image Reconstruction

After electrophysiological recording, slices were immediately fixed in a fixative containing 4% paraformaldehyde and 15% picric acid in 0.1 M phosphate buffer (PB; pH = 7.4) at 4°C for at least 12 h, then stored in 0.1 M PB with 0.05% sodium azide as a preservative at 4°C. Slices were embedded in 10% gelatin and further sectioned into slices of 50 μm thickness in cold PB using a vibratome VT1000S (Leica Microsystems, UK). After sectioning, the slices were rinsed in 0.1 M PB (3 x 10 min) and cryoprotected: first step in 10% (30 min), and later in 20% sucrose (1 h) dissolved in PB and then permeabilized using a freeze and thaw procedure. Finally, they were incubated in fluorophore (Cy3)-conjugated streptavidin (1:400, Jackson ImmunoResearch Lab.Inc. US) in 0.1 M Tris-buffered saline (TBS, pH 7.4) for 2.5 h (at 22–24°C). After washing with 0.1 M PB (3 x 10 min), the sections were covered in Vectashield mounting medium (Vector Laboratories Inc, US), put under cover slips, and examined under epifluorescence microscope (Leica DM 5000 B, UK). Sections selected for immunohistochemistry and cell reconstruction were dismounted and processed as explained below.

Some sections for cell structure illustrations were further incubated in a solution of conjugated avidin-biotin horseradish peroxidase (ABC; 1:300; Vector Labs, UK) in Tris-buffered saline (TBS, pH = 7.4) at 4°C overnight. The enzyme reaction was revealed by the glucose oxidase-DAB-nickel method using 3’3-diaminobenzidine tetrahydrochloride (0.05%) as chromogen and 0.01% H_2_O_2_ as oxidant. Sections were postfixed with 1% OsO_4_ in 0.1M PB. After several washes in distilled water, sections were stained in 1% uranyl acetate and dehydrated in ascending series of ethanol. Sections were infiltrated with epoxy resin (Durcupan) overnight and embedded on glass slices. Three-dimensional light microscopic reconstructions from sections were carried out using the Neurolucida system with 100 x objective (Olympus BX51, Olympus UPlanFI, Hungary). Images were collapsed in *z*-axis for illustration. Cells in Fig 1 were reconstructed from confocal microscope *z*-stack images of streptavidin fluorophore signal using Image-J software as described previously [32]. Vgat immunoreaction analysis was used in parallel to confirm the interneuron axon.

### Immunohistochemistry

For immunohistological reactions, free-floating sections were washed 3 times in TBS-TX 0.3% (15 min) at 22–24°C, then moved in 20% blocking solution with horse serum in TBS-TX 0.3%. The sections were incubated in primary antibodies diluted in 1% serum in TBS-TX 0.3% over three nights at 4°C, then put in relevant fluorochrome-conjugated secondary antibodies in 1% of blocking serum in TBS-TX 0.3% overnight at 4°C. Sections were washed at first step in TBS-TX 0.3% (3 x 20 min) and later in 0.1 M PB (3 x 20 min) and mounted on glass slides with Vectashield mounting medium (Vector Lab.Inc., UK). The characterizations of antibodies: pv (goat anti-pv, 1:500, Swant, Switzerland, www.swant.com), sst (rat anti-sst, 1:50, Merck Millipore, Germany, www.merckmillipore.com) and vgat (rabbit anti-vgat, 1:500, Synaptic Systems, Germany, www.sysy.com). Fluorophore-labelled secondary antibodies were: DyLight 488 (Donkey anti goat, 1:400, Jackson ImmunoResearch Lab. Inc., www.jacksonimmuno.com, US), Alexa488 (Donkey anti rat, 1:400, Jackson ImmunoResearch Lab. Inc.) and Cy5 (Donkey anti rabbit, 1:500, Jackson ImmunoResearch Lab. Inc.). Labelling of neurons by neurobiotin and immunoreactions were evaluated using first epifluorescence (Leica DM 5000 B, UK) and then laser scanning confocal microscopy (Olympus FV1000, Hungary). Immunoreaction was considered to be negative when fluorescence was not detected in relevant neurobiotin-labelled cell, but immunopositivity was detected in the same area in unlabelled cells. Immunoreactions were studied in axon boutons (vgat and pv) and soma and dendrites (pv, sst).

## Supporting Information

S1 Fig LTD is generated in PC-FSIN pairs with VLEs. The data include all FSINs stimulated with 40 Hz in control conditions at resting membrane potential of the postsynaptic cell (n = 10). The LTD fails in pairs with small EPSP. Plot shows the average baseline EPSP amplitude (failures excluded) versus the baseline-normalized EPSP amplitude(failures included) after (20–25 min) the 40 Hz presynaptic bursts (ordinate). Cells with significant LTD (studied with paired *t*-test in each experiment) are illustrated with open symbols (n = 5). Cells not showing LTD are shown with solid symbols (n = 5). Linear regression demonstrates a relation (r^2^ = 0.75) and Pearson’s test shows strong correlation (*P* < 0.001) between the baseline EPSP amplitude and the level of LTD. The amplitudes of the LTD cells and the non-LTD cells are significantly different in baseline (*P* < 0.01, Mann-Whitney test) (S7 Data).
(TIF)

S2 Fig Single fiber connections from pyramidal cells to non–fast-spiking vgat+ interneurons (nonFSIN) with large amplitude EPSP can show LTD. (A) (A1) Partial reconstruction of a synaptically connected pyramidal cell (soma and dendrites red, axon orange)–non-fast spiking interneuron (blue, axon light blue) pair visualized with streptavidin-DAB and reconstructed from one 60 μm -thick section. Schematic shows experimental design. L1 = layer 1, L2-3 = layers 2–3, separated by grey line. Scale 50 μm. (A2) Single EPSPs from the PC trigger action potentials in the postsynaptic interneuron in resting membrane potential (Em -69 mV). Compared to the fast-spiking cell, the interneuron fires with long and variable delay to the PC spike (see Fig 4A). The action potential positive peaks are marked in the abscissa. Note also the slow spike waveform. (A3) PC spike (red) and large amplitude EPSC (blue, average of 5) in the same connection in voltage-clamp (at -60 mV). Scales 1 nA and 200 pA / 5 ms. (A4) Confocal micrographs show neurobiotin (nb, top) -filled axon of the postsynaptic cell with positive immunoreaction for vgat in boutons (middle). Arrows in merged images (bottom) show co-labelled boutons (scale 5 μm). (B) (B1) EPSP amplitude in the same synaptic pair (110615_1) before and after pyramidal cell 40 Hz bursts (arrow at 0-time point). EPSP amplitude depression at 20–25 min (*P* < 0.001, paired *t*-test) is preceded by a transient enhancement of the EPSP. Postsynaptic EPSPs (blue, average of 5 at Em -68 mV) and presynaptic spikes (red) shown on top at time points indicated. One presynaptic 40 Hz burst illustrated. (B2) Similar experiment with another postsynaptic non-fast spiking interneuron with large EPSP, but now in the presence of mGluR blockers LY367385 (100 μM) and MPEP (25 μM). Unlike in FSINs (see Fig 2A and 2B), the EPSP amplitude shows significant depression (at 20–25 min, *P* < 0,001, paired -test) in the presence of the drugs (S8 Data).
(TIF)

S3 Fig Monosynaptic IPSCs from FSIN to PC have submillisecond delay to the presynaptic spike. Onsets of the monosynaptic IPSCs from FSINs to pyramidal cells exhibit submillisecond (average 0.96 ± 0.10 ms, n = 9,mean ± s.e.m) delay to the presynaptic cell spike. This is significantly shorter than the onset delay of dIPSCs (6.23 ± 0.72 ms, n = 16 pairs, *P* < 0.001, *t*-test). Schematic on top illustrates experimental design. Traces on top are from one cell pair (blue, FSIN spike) showing 5 superimposed consecutive monosynaptic IPSCs (red) in the postsynaptic pyramidal cell. Plot below shows mean ± s.e.m. of the monosynaptic IPSC (n = 20 in each cell) onset delay in the FSIN—PC cell pairs (experiment codes indicated in the ordinate) (S9 Data).
(TIF)

S4 Fig The dIPSCs evoked by single PC spike are blocked with ionotropic glutamate AMPA receptor antagonist GYKI53655. (A) Raster plot of single PC spike -evoked dIPSC onset delay in one experiment. AMPAR blocker GYKI53655 (25 μM) was applied at 0 -time point (dotted horizontal line). Traces above show consecutive dIPSCs (blue) in the postsynaptic pyramidal cell during the baseline and in the presence of GYKI (5 min). Schematic inset shows experimental design with pre- (PC1) and postsynaptic pyramidal cell (PC2) for the dIPSCs. (B) dIPSC onset delay histograms in three experiments (shown as pairs 1, 2 and 3) showing probability and delay of the first dIPSC evoked by PC spike. After baseline, the dIPSCs are blocked by GYKI. The pair 1 is the same experiment as shown in the raster plot (S6 Data).
(TIF)

S5 Fig Amplitude of the dIPSCs is similar to the amplitude of monosynaptic IPSCs from FSINs to PCs (t -test). The plot shows in left the average amplitudes of monosynaptic IPSCs (monIPSCs) elicited in 9 FSIN–PC pairs (failures excluded) as reported in the results. Average amplitudes of the disynaptic IPSCs (dIPSCs, failures excluded) shown in right. This suggests the earliest complex event IPSCs with 6.23 ± 0.72 ms delay are generated by single fast-spiking interneurons. Plot shows mean ± s.e.m. of the amplitudes in baseline conditions (S10 Data).
(TIF)

S1 Table Results on EPSP/EPSC analyses and immunohistochemical reactions of the three postsynaptic cell populations: fast-spiking interneurons (FSINs), non–fast-spiking interneurons (nonFSINs) and pyramidal cells (PCs). The table comprises EPSP data in baseline conditions for 31 postsynaptic vgat+ cells studied for long-term plasticity and 16 PCs in the triple- and paired recordings. Experiment code identifies the postsynaptic cell recorded. Ten fast-spiking putative basket cells were anatomically identified. EPSP average amplitude is shown without synaptic failures and including the failures. Experiments are listed in a descending order starting from cells with the largest average EPSPs. EPSP failure rate is a percentage of failures of the EPSP to the presynaptic spike. EPSP rise time (from 20% to 80% of maximum) was measured from averaged EPSPs. Paired-pulse ratio (PPR) is defined as 1^st^ EPSP / 2^nd^ EPSP amplitude. EPSCs represent an average of 5 responses recorded in first 2 minute period in the voltage-clamp after break-in to the whole cell mode from the giga-seal. Because the EPSCs were recorded immediately after break-in (when whole-cell properties are typically not yet stabilized), the measured values may represent underestimate of the current. Note that in 3 FSINs and in one PC, EPSCs were not measured. Immunohistochemical reactions for vesicular GABA transporter (vgat), parvalbumin (pv) and somatostatin (sst) are indicated by (+) for positive and (-) for negative. The vgat was analyzed in axon terminals, pv in axon terminals, soma or dendrites, and sst in soma and dendrites. Putative basket cells in the FSINs were identified by their axon forming collaterals around L2-3 neuron somatae. No axo-axonic cell types were identified. Pyramidal cells showed characteristic densely spiny dendrites with filopodial, stubby and mushroom-shaped spines, and main axon projecting towards infragranular layers. In the PCs table “a” and “b” indicate distinct postsynaptic cells in a mutually connected PC couple.
(DOCX)

S1 Data Data for Fig 1B1–1B3. (XLSX)

S2 Data Data for Fig 2A2–2A5. (XLSX)

S3 Data Data for Fig 2B1 and 2B2. (XLSX)

S4 Data Data for Fig 3A2–3A5, 3B1 and 3B2. (XLSX)

S5 Data Data for Fig 3C2–3C5. (XLSX)

S6 Data Data for Fig 4B2, 4D–4I and S4 Fig. (XLSX)

S7 Data Data for S1 Fig. (XLSX)

S8 Data Data for S2B1 and S2B2 Fig. (XLSX)

S9 Data Data for S3 Fig. (XLSX)

S10 Data Data for S5 Fig. (XLSX)

## Abbreviations

AP: action potential
CV: coefficient of variation
dIPSC: disynaptic GABA_A_R-mediated inhibitory current
DL-APV: DL-2-Amino-5-phosphonopentanoic acid
EPSC: excitatory postsynaptic current
EPSP: excitatory postsynaptic potential
FSIN: fast-spiking GABAergic interneuron
LTD: long-term depression
mGluR: metabotropic glutamate receptor
monIPSC: monosynaptic GABA_A_R-mediated inhibitory current
MPEP: 2-Methyl-6-(phenylethynyl)pyridine hydrochloride
NMDAR: *N*-methyl-D-aspartate receptor
non-FSIN: non–fast-spiking GABAergic interneuron
IPSC: inhibitory postsynaptic current
PC: pyramidal cell
PPR: paired-pulse ratio
pv: parvalbumin
SD: standard deviation
s.e.m.: standard error of the mean
sst: somatostatin
SW: spike inward current width
vgat: vesicular GABA transporter
VLE: very large glutamatergic EPSP
