# Supplementary figures and images for: Plasticity in Single Axon Glutamatergic Connection to GABAergic Interneurons Regulates Complex Events in the Human Neocortex
Source: PLoS Biol. 2016 Nov 9;14(11):e2000237. doi: 10.1371/journal.pbio.2000237 (PMC5102409; doi:10.1371/journal.pbio.2000237)

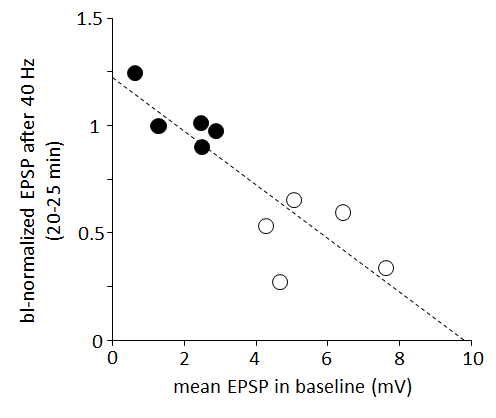

Supplement: S1 Fig — The data include all FSINs stimulated with 40 Hz in control conditions at resting membrane potential of the postsynaptic cell (n = 10). The LTD fails in pairs with small EPSP. Plot shows the average baseline EPSP amplitude (failures excluded) versus the baseline-normalized EPSP amplitude(failures included) after (20–25 min) the 40 Hz presynaptic bursts (ordinate). Cells with significant LTD (studied with paired t-test in each experiment) are illustrated with open symbols (n = 5). Cells not showing LTD are shown with solid symbols (n = 5). Linear regression demonstrates a relation (r2 = 0.75) and Pearson’s test shows strong correlation (P < 0.001) between the baseline EPSP amplitude and the level of LTD. The amplitudes of the LTD cells and the non-LTD cells are significantly different in baseline (P < 0.01, Mann-Whitney test) (S7 Data). (TIF) [file pbio.2000237.s001.tif]

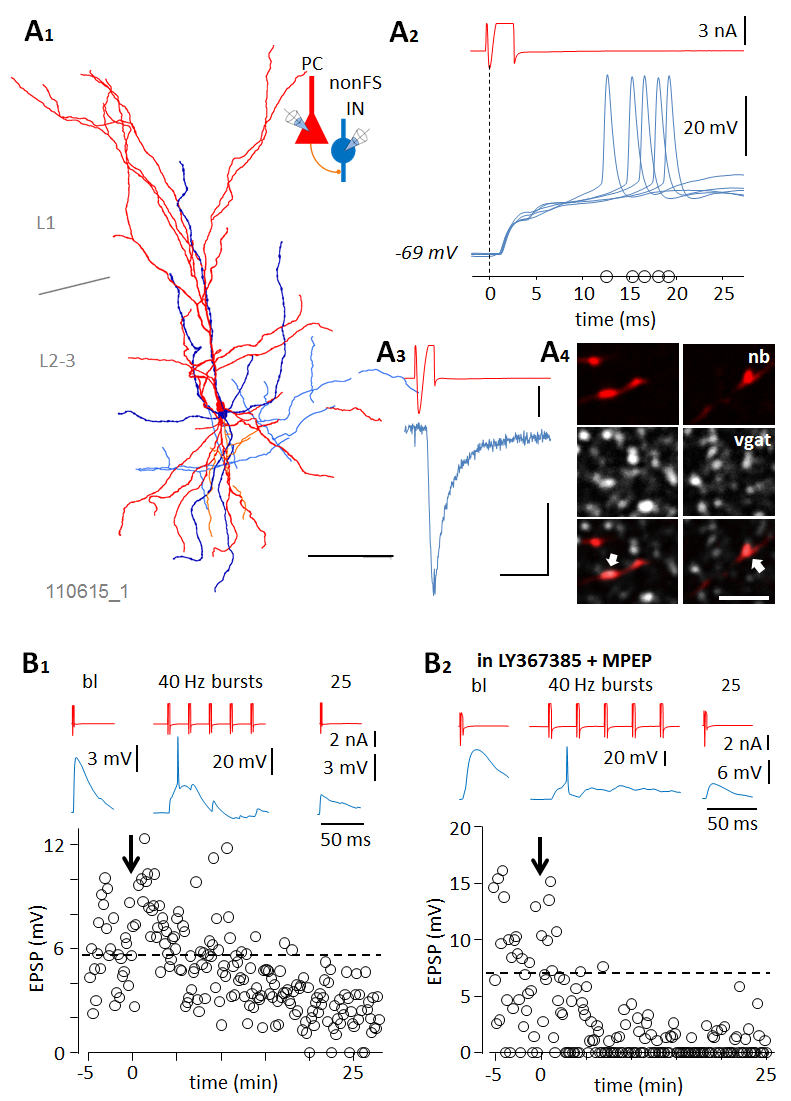

Supplement: S2 Fig — (A) (A1) Partial reconstruction of a synaptically connected pyramidal cell (soma and dendrites red, axon orange)–non-fast spiking interneuron (blue, axon light blue) pair visualized with streptavidin-DAB and reconstructed from one 60 μm -thick section. Schematic shows experimental design. L1 = layer 1, L2-3 = layers 2–3, separated by grey line. Scale 50 μm. (A2) Single EPSPs from the PC trigger action potentials in the postsynaptic interneuron in resting membrane potential (Em -69 mV). Compared to the fast-spiking cell, the interneuron fires with long and variable delay to the PC spike (see Fig 4A). The action potential positive peaks are marked in the abscissa. Note also the slow spike waveform. (A3) PC spike (red) and large amplitude EPSC (blue, average of 5) in the same connection in voltage-clamp (at -60 mV). Scales 1 nA and 200 pA / 5 ms. (A4) Confocal micrographs show neurobiotin (nb, top) -filled axon of the postsynaptic cell with positive immunoreaction for vgat in boutons (middle). Arrows in merged images (bottom) show co-labelled boutons (scale 5 μm). (B) (B1) EPSP amplitude in the same synaptic pair (110615_1) before and after pyramidal cell 40 Hz bursts (arrow at 0-time point). EPSP amplitude depression at 20–25 min (P < 0.001, paired t-test) is preceded by a transient enhancement of the EPSP. Postsynaptic EPSPs (blue, average of 5 at Em -68 mV) and presynaptic spikes (red) shown on top at time points indicated. One presynaptic 40 Hz burst illustrated. (B2) Similar experiment with another postsynaptic non-fast spiking interneuron with large EPSP, but now in the presence of mGluR blockers LY367385 (100 μM) and MPEP (25 μM). Unlike in FSINs (see Fig 2A and 2B), the EPSP amplitude shows significant depression (at 20–25 min, P < 0,001, paired -test) in the presence of the drugs (S8 Data). (TIF) [file pbio.2000237.s002.tif]

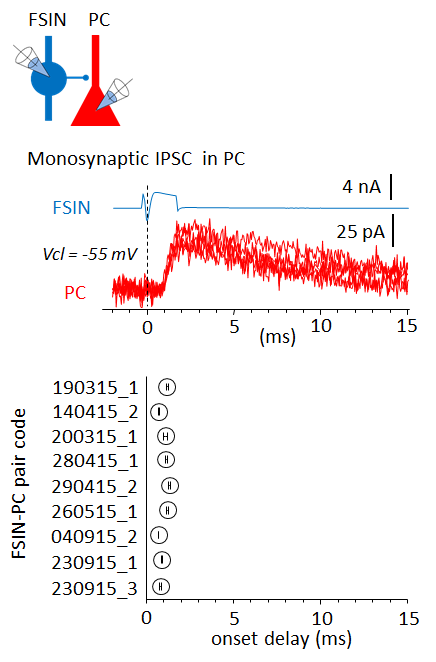

Supplement: S3 Fig — Onsets of the monosynaptic IPSCs from FSINs to pyramidal cells exhibit submillisecond (average 0.96 ± 0.10 ms, n = 9,mean ± s.e.m) delay to the presynaptic cell spike. This is significantly shorter than the onset delay of dIPSCs (6.23 ± 0.72 ms, n = 16 pairs, P < 0.001, t-test). Schematic on top illustrates experimental design. Traces on top are from one cell pair (blue, FSIN spike) showing 5 superimposed consecutive monosynaptic IPSCs (red) in the postsynaptic pyramidal cell. Plot below shows mean ± s.e.m. of the monosynaptic IPSC (n = 20 in each cell) onset delay in the FSIN—PC cell pairs (experiment codes indicated in the ordinate) (S9 Data). (TIF) [file pbio.2000237.s003.tif]

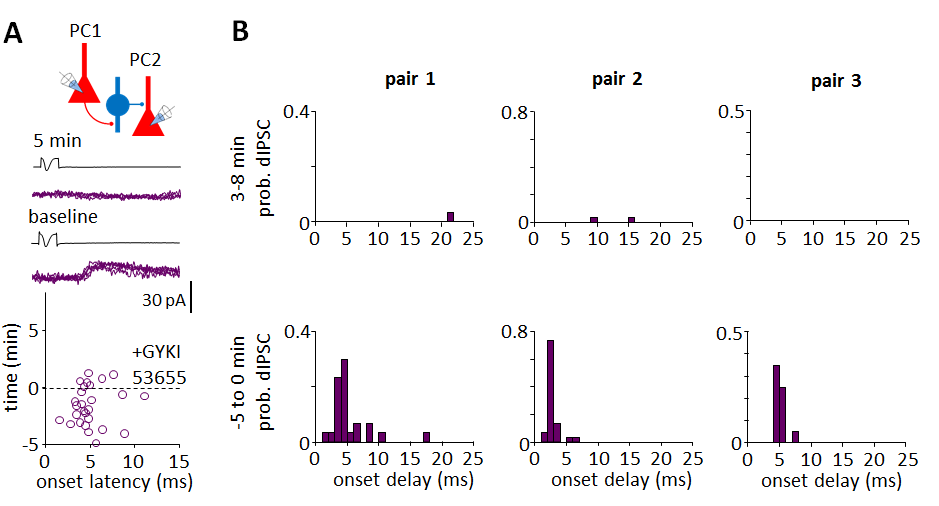

Supplement: S4 Fig — (A) Raster plot of single PC spike -evoked dIPSC onset delay in one experiment. AMPAR blocker GYKI53655 (25 μM) was applied at 0 -time point (dotted horizontal line). Traces above show consecutive dIPSCs (blue) in the postsynaptic pyramidal cell during the baseline and in the presence of GYKI (5 min). Schematic inset shows experimental design with pre- (PC1) and postsynaptic pyramidal cell (PC2) for the dIPSCs. (B) dIPSC onset delay histograms in three experiments (shown as pairs 1, 2 and 3) showing probability and delay of the first dIPSC evoked by PC spike. After baseline, the dIPSCs are blocked by GYKI. The pair 1 is the same experiment as shown in the raster plot (S6 Data). (TIF) [file pbio.2000237.s004.tif]

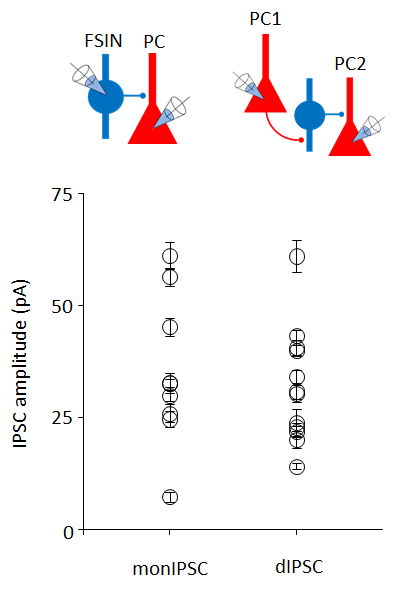

Supplement: S5 Fig — The plot shows in left the average amplitudes of monosynaptic IPSCs (monIPSCs) elicited in 9 FSIN–PC pairs (failures excluded) as reported in the results. Average amplitudes of the disynaptic IPSCs (dIPSCs, failures excluded) shown in right. This suggests the earliest complex event IPSCs with 6.23 ± 0.72 ms delay are generated by single fast-spiking interneurons. Plot shows mean ± s.e.m. of the amplitudes in baseline conditions (S10 Data). (TIF) [file pbio.2000237.s005.tif]
