# Supplementary material for: Plasticity in Single Axon Glutamatergic Connection to GABAergic Interneurons Regulates Complex Events in the Human Neocortex
Source: PLoS Biol. 2016 Nov 9;14(11):e2000237. doi: 10.1371/journal.pbio.2000237 (PMC5102409; doi:10.1371/journal.pbio.2000237)
Supplement: S1 Table — The table comprises EPSP data in baseline conditions for 31 postsynaptic vgat+ cells studied for long-term plasticity and 16 PCs in the triple- and paired recordings. Experiment code identifies the postsynaptic cell recorded. Ten fast-spiking putative basket cells were anatomically identified. EPSP average amplitude is shown without synaptic failures and including the failures. Experiments are listed in a descending order starting from cells with the largest average EPSPs. EPSP failure rate is a percentage of failures of the EPSP to the presynaptic spike. EPSP rise time (from 20% to 80% of maximum) was measured from averaged EPSPs. Paired-pulse ratio (PPR) is defined as 1st EPSP / 2nd EPSP amplitude. EPSCs represent an average of 5 responses recorded in first 2 minute period in the voltage-clamp after break-in to the whole cell mode from the giga-seal. Because the EPSCs were recorded immediately after break-in (when whole-cell properties are typically not yet stabilized), the measured values may represent underestimate of the current. Note that in 3 FSINs and in one PC, EPSCs were not measured. Immunohistochemical reactions for vesicular GABA transporter (vgat), parvalbumin (pv) and somatostatin (sst) are indicated by (+) for positive and (-) for negative. The vgat was analyzed in axon terminals, pv in axon terminals, soma or dendrites, and sst in soma and dendrites. Putative basket cells in the FSINs were identified by their axon forming collaterals around L2-3 neuron somatae. No axo-axonic cell types were identified. Pyramidal cells showed characteristic densely spiny dendrites with filopodial, stubby and mushroom-shaped spines, and main axon projecting towards infragranular layers. In the PCs table “a” and “b” indicate distinct postsynaptic cells in a mutually connected PC couple. (DOCX) [file pbio.2000237.s006.docx]

| **Experiment code** | **EPSP average amplitude (mV) failures excluded** | **EPSP average amplitude (mV) with failures** | **1^st^ EPSP failure rate (%)** | **EPSP rise time 20-80% (ms)** | **PPR 1^st^ vs. 2^nd^** | **EPSC time to peak (ms)** | **Average EPSC ampl. (pA)** | **Immunohistochemistry** | **Cell identity** |
| --- | --- | --- | --- | --- | --- | --- | --- | --- | --- |
| **FSINs** | | | | | | | | | |
| 140416_2 | 16.49 | 16.49 | 0.00 | 1.00 | 0.88 | 0.98 | 310.00 | vgat+ |  |
| 050416_1 | 9.65 | 9.65 | 0.00 | 0.95 | 1.54 | 0.50 | 410.00 | vgat+ pv+ |  |
| 260216_2 | 8.05 | 8.05 | 0.00 | 1.12 | 1.35 |  |  | vgat+ |  |
| 040315_3 | 7.65 | 7.65 | 0.00 | 0.85 | 2.12 | 0.76 | 130.00 | vgat+ pv+ |  |
| 290415_2 | 6.44 | 6.44 | 0.00 | 0.28 | 0.96 | 0.42 | 347.80 | vgat+ |  |
| 200315_1 | 5.96 | 5.96 | 0.00 | 0.52 | 2.45 | 0.61 | 152.00 | vgat+ pv+ |  |
| 250615_5 | 5.06 | 5.06 | 0.00 | 0.56 | 1.37 | 0.74 | 147.70 | vgat+ pv+ |  |
| 280815_1 | 4.69 | 4.69 | 0.00 | 0.62 | 1.41 |  |  | vgat+ pv+ | put. basket |
| 230915_3 | 4.47 | 4.47 | 0.00 | 2.06 | 1.69 | 0.68 | 163.00 | vgat+ | put. basket |
| 140415_2 | 4.29 | 4.29 | 0.00 | 0.87 | 1.59 |  |  | vgat+ | put. basket |
| 161015_1 | 2.92 | 2.72 | 7.00 | 0.44 | 1.17 | 0.35 | 77.80 | vgat+ pv+ | put. basket |
| 250615_3 | 2.50 | 2.50 | 0.00 | 1.02 | 1.66 | 0.78 | 39.40 | vgat+ pv+ |  |
| 030415_1 | 2.46 | 2.46 | 0.00 | 1.77 | 1.59 | 0.90 | 30.73 | vgat+ pv+ |  |
| 050615_1 | 2.45 | 2.11 | 17.00 | 0.48 | 1.35 | 0.43 | 70.60 | vgat+ pv+ |  |
| 040915_2 | 1.91 | 1.86 | 4.00 | 0.67 | 1.30 | 0.40 | 40.40 | vgat+ | put. basket |
| 170915_2 | 1.56 | 1.51 | 4.00 | 0.70 | 1.02 | 0.38 | 77.40 | vgat+ pv+ | put. basket |
| 120815_3 | 1.29 | 1.14 | 17.00 | 0.60 | 1.03 | 0.59 | 33.10 | vgat+ | put. basket |
| 250615_1 | 1.28 | 0.81 | 49.00 | 0.51 | 1.25 | 0.64 | 33.70 | vgat+ pv+ |  |
| 230915_2 | 1.18 | 1.17 | 7.00 | 0.55 | 1.13 | 0.47 | 28.10 | vgat+ | put. basket |
| 260515_3 | 0.87 | 0.84 | 3.00 | 0.94 | 1.14 |  |  | vgat+ |  |
| 230915_1 | 0.85 | 0.70 | 26.00 | 0.42 | 1.36 | 0.40 | 70.20 | vgat+ | put. basket |
| 280415_1 | 0.62 | 0.62 | 0.00 | 1.94 | 1.46 | 0.66 | 10.22 | vgat+ | put. basket |
| **non-FSINs** | | | | | | | |  |  |
| 100316_1 | 6.89 | 6.74 | 13.00 | 6.51 | 0.97 | 0.90 | 290.00 | vgat+ |  |
| 110615_1 | 5.81 | 5.81 | 0.00 | 1.90 | 0.84 | 0.45 | 132.00 | vgat+ |  |
| 281015_1 | 3.26 | 3.20 | 0.00 | 1.62 | 0.99 | 1.93 | 56.00 | vgat+ sst- |  |
| 150415_2 | 3.25 | 3.25 | 0.00 | 1.58 | 0.74 | 1.80 | 58.00 | vgat+ |  |
| 280815_2 | 3.17 | 3.17 | 5.00 | 1.59 | 2.19 | 1.29 | 29.80 | vgat+ sst- |  |
| 280415_2 | 1.53 | 1.17 | 30.00 | 1.96 | 0.89 | 2.40 | 38.00 | vgat+ |  |
| 190315_1 | 0.90 | 0.90 | 0.00 | 1.96 | 1.28 | 0.60 | 41.60 | vgat+ |  |
| 040915_1 | 0.87 | 0.58 | 33.00 | 1.65 | 1.25 | 0.97 | 13.60 | vgat+ |  |
| 280415_3 | 0.71 | 0.71 | 0.00 | 2.22 | 1.17 | 2.00 | 7.20 | vgat+ |  |
| **PCs** | | | | | | | |  |  |
| 281015_2 | 3.26 | 3.26 | 0.00 | 2.50 | 1.03 | 2.70 | 58.60 |  |  |
| 150415_3 | 3.24 | 3.24 | 0.00 | 2.40 | 0.74 | 1.93 | 114.10 |  |  |
| 120315_1 cell b | 2.71 | 2.71 | 0.00 | 2.07 | 1.19 | 1.87 | 68.70 |  |  |
| 260515_2 | 2.67 | 2.67 | 0.00 | 1.45 | 1.19 | 1.80 | 51.00 |  |  |
| 110615_2 | 2.60 | 1.68 | 37.00 | 3.02 | 0.80 | 1.46 | 24.40 |  |  |
| 170915_3 cell a | 2.49 | 2.49 | 0.00 | 1.23 | 1.16 | 1.17 | 30.40 |  |  |
| 031115_1 | 2.27 | 2.27 | 0.00 | 1.60 | 1.07 | 1.71 | 59.00 |  |  |
| 290415_5 | 2.15 | 1.45 | 37.00 | 1.50 | 0.94 | 0.77 | 56.80 |  |  |
| 021015_1 | 2.11 | 1.19 | 37.00 | 2.10 | 0.91 |  |  |  |  |
| 210515_1 | 1.82 | 1.82 | 0.00 | 2.94 | 1.08 | 2.63 | 16.60 |  |  |
| 120315_1 cell a | 1.63 | 1.63 | 0.00 | 1.92 | 0.87 | 2.01 | 80.00 |  |  |
| 260216_1 | 1.29 | 1.23 | 7.00 | 1.77 | 1.02 | 2.78 | 51.00 |  |  |
| 170415_1 | 1.14 | 1.14 | 0.00 | 1.55 | 1.14 | 1.76 | 19.70 |  |  |
| 030316_1 | 1.10 | 1.10 | 0.00 | 2.20 | 1.40 | 2.70 | 21.00 |  |  |
| 040315_1 | 0.96 | 0.96 | 0.00 | 1.30 | 0.99 | 1.43 | 18.00 |  |  |
| 040316_1 | 0.75 | 0.70 | 7.00 | 1.70 | 1.14 | 2.20 | 12.00 |  |  |
